# Management of giant left atrial thrombus late after transcatheter mitral valve-in-ring replacement using a transcatheter aortic valve: a case report

**DOI:** 10.1186/s12872-024-04260-9

**Published:** 2024-11-13

**Authors:** A. Maloku, A. Hamadanchi, L. Bäz, M. Richter, S. Bargenda, S. Möbius-Winkler, P. C. Schulze, Marcus Franz

**Affiliations:** 1 Department of Internal Medicine I, Cardiology, Angiology, Intensive Medical Care, University Hospital Jena, Am Klinikum 1, 07747 Jena, Germany; 2 Department of Cardiothoracic Surgery, University Hospital Jena, Am Klinikum 1, 07747 Jena, Germany; 3 Department of Cardiology, Angiology and Intensive Care Medicine, Cardiovascular Center Rotenburg, Klinikum Hersfeld-Rotenburg, Heinz-Meise-Straße 100, 36199 Rotenburg an der Fulda, Germany

**Keywords:** Left atrial thrombus, Transcatheter mitral valve replacement, Oral anticoagulation

## Abstract

**Background:**

In symptomatic high-risk patients with severe mitral valve regurgitation (MR), who are not eligible for surgery, Transcatheter edge-to-edge repair (TEER) or transcatheter mitral valve replacement (TMVR) may be an option, especially when surgical mitral valve repair by annuloplasty has been performed earlier. After TMVR, the appropriate anticoagulation regimen is still matter of debate.

**Case presentation:**

We here report on a 78-year-old frail lady with heart failure and atrial fibrillation who underwent surgical reconstruction of the mitral valve nine years ago. Due to high surgical risk and after heart team discussion, TMVR using a transcatheter aortic valve prosthesis (valve-in-ring concept) was performed successfully via the transapical access route. Several months later, an excellent result could be confirmed. Since surgical excision of the left atrial appendage was carried out during first surgery, oral anticoagulation was withdrawn. Two months later, the patient presented with a massive LA thrombus mass and severe stenosis of the mitral valve prosthesis requiring re-do surgery.

**Conclusions:**

Management of anticoagulation in patients with atrial fibrillation and successfully performed LAA excision is still a matter of debate, in particular after transcatheter heart valve implantation in mitral position. TMVR devices may be very thrombogenic. Thus, caution should be used whenever discontinuing oral anticoagulation in these patients. Despite the lack of evidence, withdrawal of anticoagulation should be avoided here, especially in the absence of bleeding complications. Left atrial appendage closure or excision should not influence this decision.

## Introduction

In symptomatic high-risk patients with severe mitral valve regurgitation (MR), who are not eligible for surgery due to multimorbidity, and further do not fulfill the criteria for a Transcatheter edge-to-edge repair (TEER) procedure, transcatheter mitral valve replacement (TMVR) may be considered (IIb C) according to current guidelines [1]. Since patients with severe MR due to failing biological mitral valve prostheses are never eligible for TEER and patients after surgical mitral valve repair are sometimes not eligible for TEER, TMVR using transcatheter aortic valve prosthesis has been successfully performed in smaller patient cohorts using both, the transapical as well as the transvascular access route. Due to that limited experiences and outcome data, there is currently no guideline recommendation for these special situations [2].

We report on a female high-risk patient who underwent successful TMVR after interdisciplinary evaluation of all treatment options with the patient as part of the heart team. While achieving an excellent clinical result for several months, the patient suffered from late development of a giant left atrial (LA) thrombus involving the transcatheter heart valve (THV) prosthesis with consecutive severe stenosis requiring re-do cardiac surgery. The latter could be performed successfully; the patient survived and is doing well.

## Case report

A 78-year-old frail lady with known atrial fibrillation (CHA_2_DS_2_-VASc Score: 7), valvular heart disease and a severely reduced systolic left ventricular function (left ventricular ejection fraction, LVEF, 25–30%), presented in a secondary care center with severe dyspnea, fatigue, cough with bloody sputum and nausea. In thorax computed tomography, pulmonary infiltrates were detected and a suspicious mass infiltrating the left atrium was recognized. For further evaluation and therapeutic management, the patient was referred to our heart center with suspected diagnosis of lymphoma or angiosarcoma of the LA.

Relevant past history included surgical reconstruction of the mitral valve by annuloplasty using a 26 mm Physioring, which was done nine years ago in 2013. Moreover, the patient underwent several ablation therapies due to symptomatic atrial fibrillation within the last years. In November 2021, a TMVR procedure using an Edwards SAPIEN 3 (ES3) 23 mm transcatheter aortic valve prosthesis had been performed due to severe symptomatic mitral valve regurgitation despite guideline-directed medial therapy. The procedure (valve-in-ring concept) was realized via the transapical access route.

The case was extensively discussed in our interdisciplinary heart team and all treatment options available (re-do surgery, medical treatment, TEER, TMVR) were considered against the background of the high surgical risk of mortality (STS Score 12.3% and Euro-Score II 52.65%). While TEER was anatomically not feasible in this lady, TMVR using a transcatheter aortic valve prosthesis concept was decided to be the best option and the patient consented to do so.

The procedure could be performed successfully, and the postoperative course was uneventful. Echocardiography in the first days after the intervention showed the prosthesis in a regular position and function with no mitral regurgitation but mild stenosis (mean pressure gradient, MPG, 10mmHg), mainly due to the relatively small ES3 23 mm prosthesis. Moreover, a severely reduced LVEF and severe tricuspid valve regurgitation remained constant. The patient could be discharged in a good condition a few days later with complete guideline directed heart failure medication for HFrEF (“fantastic four”) and apixaban as oral anticoagulation therapy.

Within the next months, regular follow-up visits in our outpatient department showed excellent clinical and echocardiographic results. The patient reported a decline in heart failure symptoms, performed well in daily life, NT-pro-BNP levels were decreasing (from 1416 pg/ml before to 1013 pg/ml at first visit after TMVR) and echocardiographic findings remained stable with good position and function of the prosthesis in mitral position not showing regurgitation and the known stenotic component with an MPG of 10 mmHg.

**Fig. 1:**
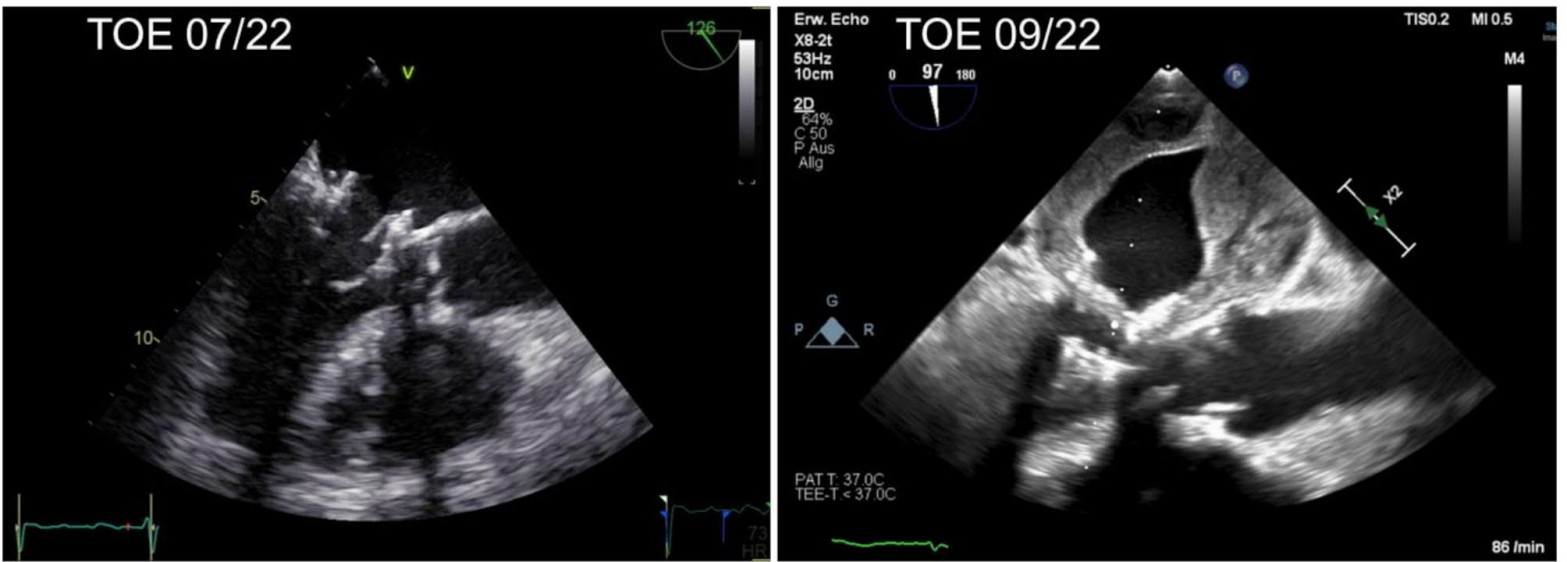
Before and after Thrombus formation

**Fig. 2:**
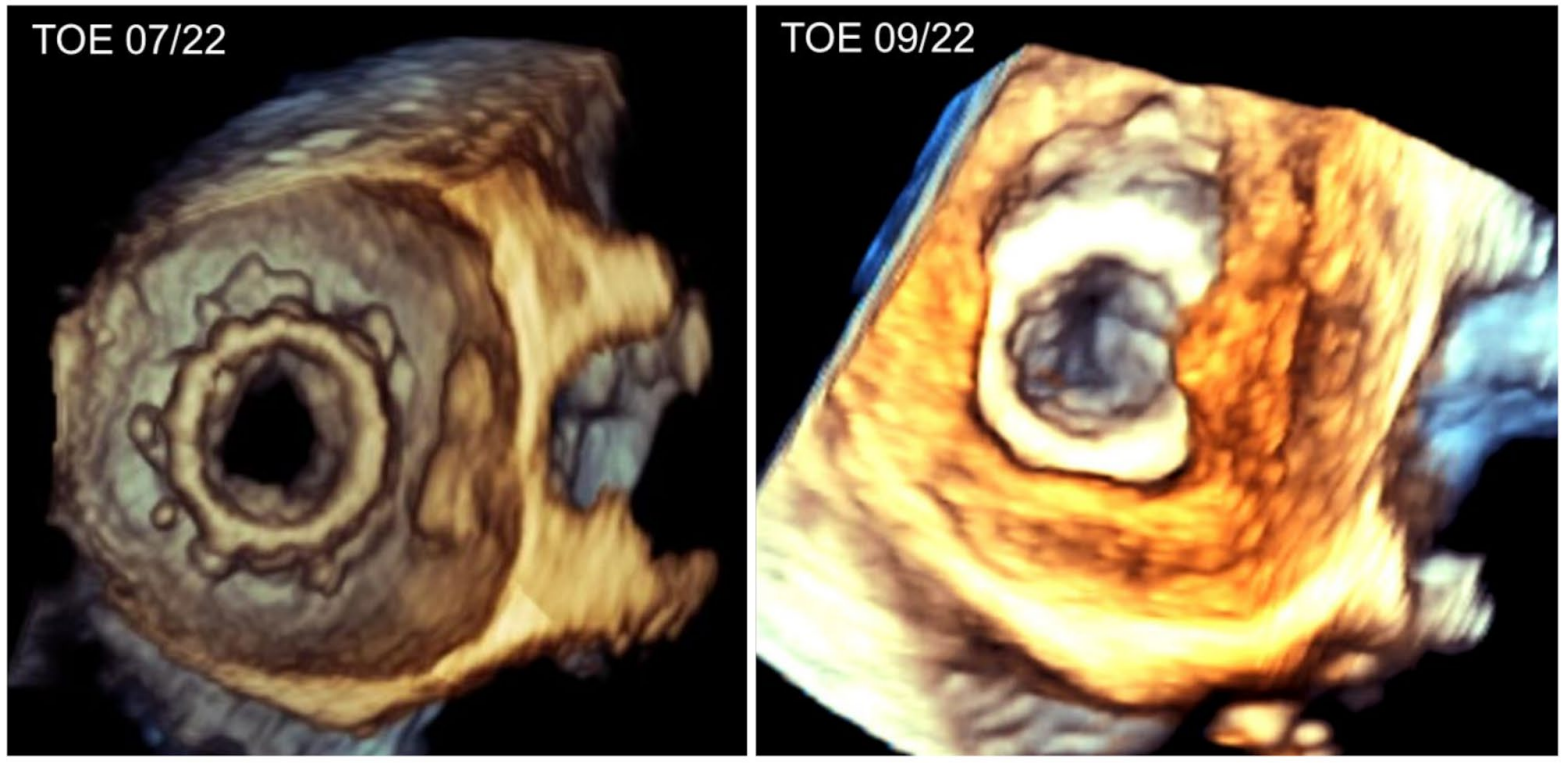
3D – Image before and after stenosis of the opening of the mitral valve

**Fig. 3:**
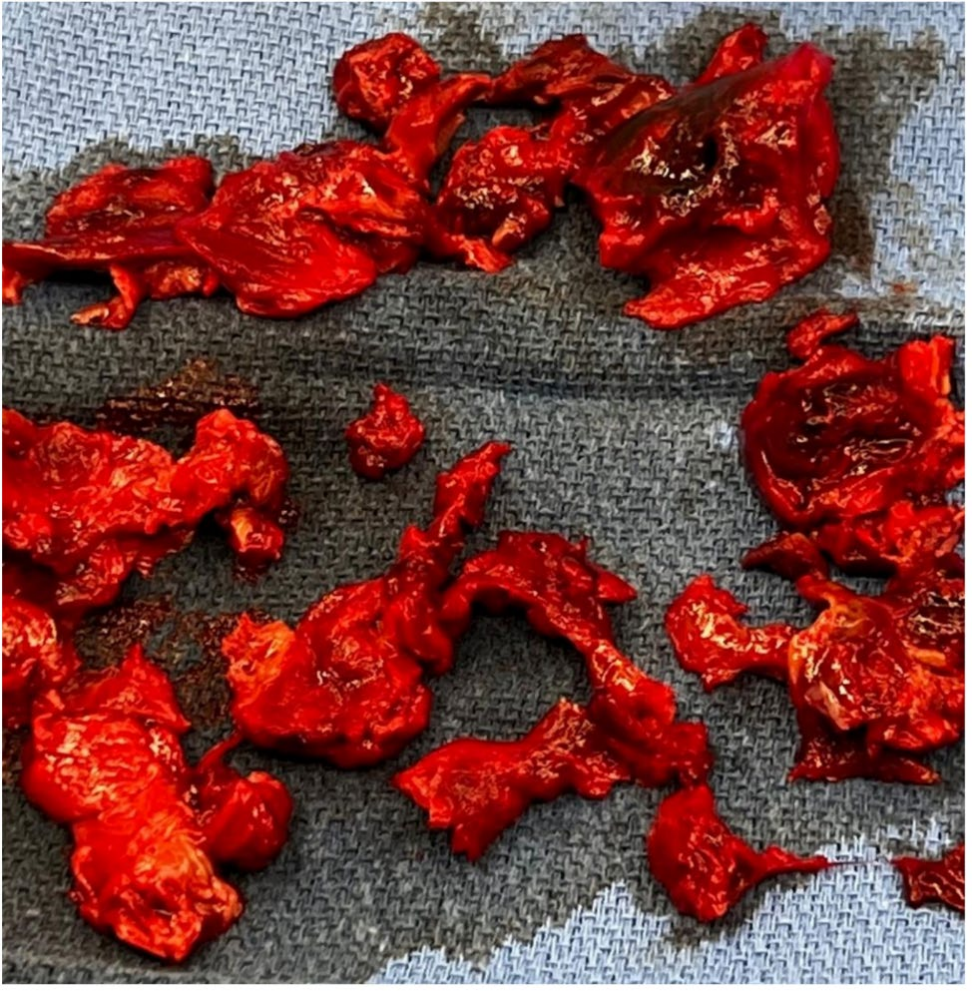
Removed thrombi from the LA

The last follow-up visit of the patient happened approximately 2 months prior to the current hospitalization in July 2022. Here, a transesophageal echocardiography (TOE) had been performed, which showed a very good long-term result with excellent function of the THV prosthesis in mitral position without any regurgitation. There were no signs of thrombus formation both, in the LA or in association with the THV prosthesis (Figs. 1, 2 and 3 on the left). The left atrial appendage (LAA), which had been closed surgically in 2013 during mitral valve repair surgery as mentioned above, revealed complete closure. Because of that finding, anticoagulation therapy was discontinued and a single antiplatelet therapy (SAPT) therapy was introduced due to atherosclerotic cardiovascular disease and elevated bleeding risk with a HAS-BLED Score of 3 points. Since more than 8 months had passed after the TMVR procedure, this was considered to be in adherence to current guidelines, which recommend that a Vitamin K antagonist should be considered for the first 3 months after the implantation of a bioprosthesis in mitral position [1]. The special situation of the patient described here is very rare and, of course, not represented by guidelines due to the lack of evidence.

**Fig. 4:**
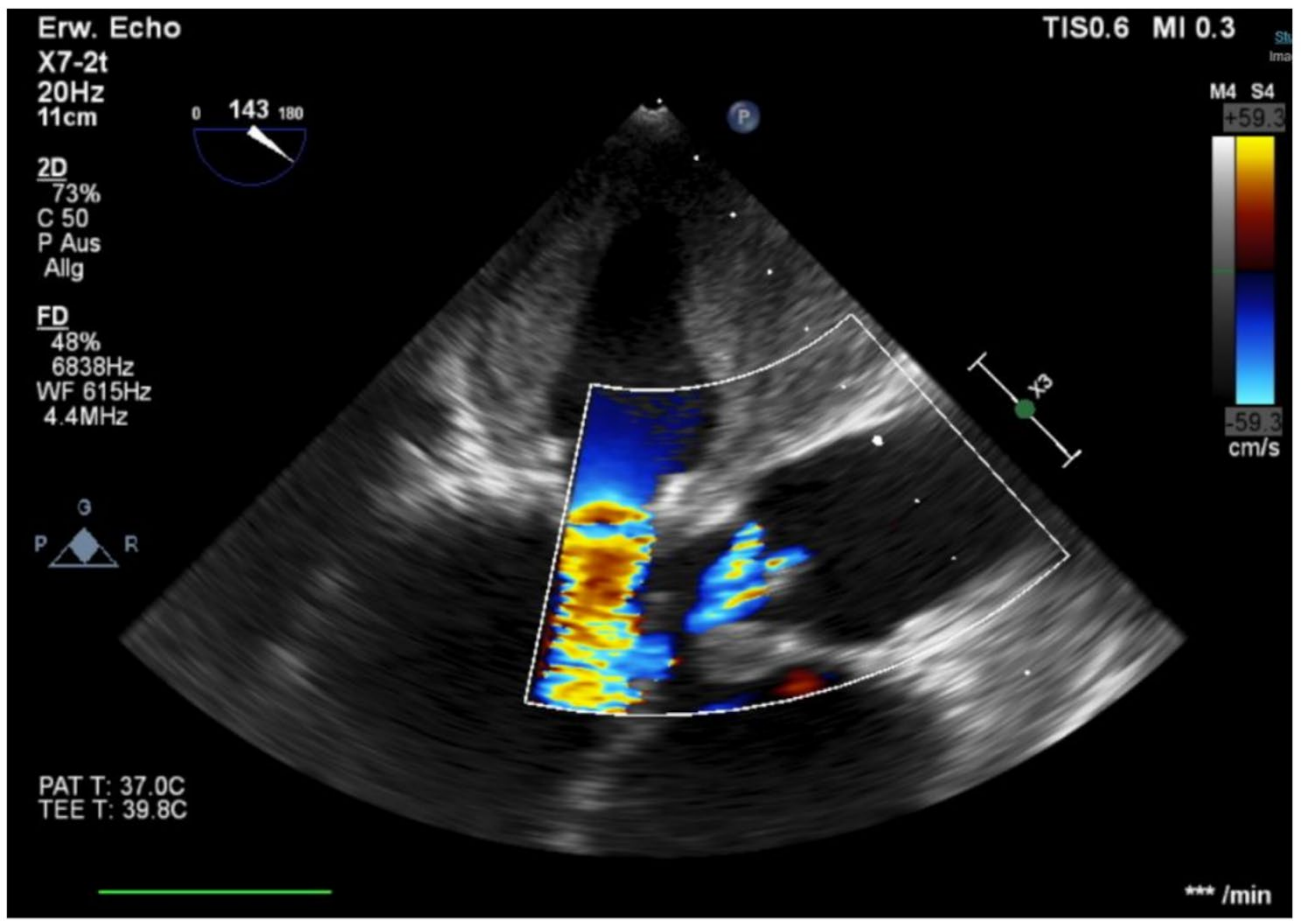
Doppler image of the mitral flow with candle flame phenomenon

**Fig. 5:**
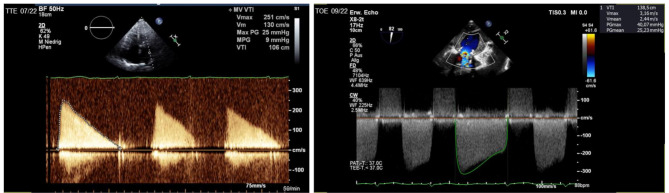
Mitral valve flow profile in CW doppler before (TTE) and after stenosis (TOE). In July.2022, there was a mean Gradient of 9 mmHg, in September 2022, there was a mean Gradient of 25 mmHg

During current hospitilization, we performed another TOE and compared the images to the one performed 2 months ago (Figs. 1, 2 and 3). This time it showed a massive LA thrombus mass, which was filling up more than 2/3 of the already dilated LA (Fig. 1, on the right). Furthermore, the ES3 23 mm prosthesis of the mitral valve appeared to be severely stenotic (Figs. 2 and 4 on the right). Using 3-dimensional (3D) TOE, we could observe a markedly reduced opening of the prosthesis (Fig. 2, left – pre stenotic; right – post stenotic). The MPG was 25 mmHg reflecting a highly increased gradient flow compared to the MPG of 10mmHg measured 2 months before (Fig. 5).

**Fig. 6:**
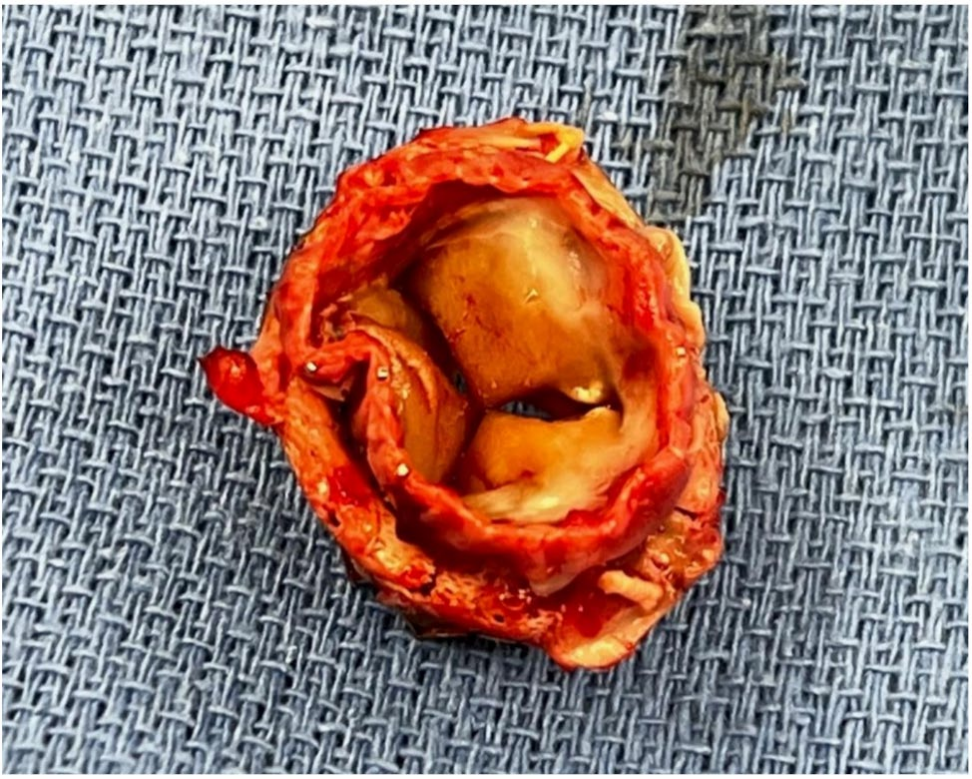
Stenotic prosthesis (ES3 23mm in 26mm Physioring)

The heart team was immediately alerted and late on the same evening, the patient was operated in terms of an ultima-ratio procedure. The entirety of the LA Thrombus was removed (Fig. 3) and the mitral valve with the stenotic THV prosthesis was excised and explanted. The explanted ES3 23 mm prosthesis did not show any signs of early valve degeneration. (Fig. 6). A biological valve (SJM-Epic, Gr. 33 mm) was implanted successfully with excellent echocardiographic results. For further management, the patient was admitted to our intensive care unit (ICU). While presenting uneventful in the first hours after surgery, the patient developed sepsis and acute kidney failure leading to the requirement of high dose catecholamines including vasopressin as well as transient hemodialysis. After 13 days of ICU treatment, the patient was transferred to normal ward. Here, unfortunately, the renal function did not improve and intermittent hemodialysis was necessary. After a time period of four weeks, the patient was discharged home in good clinical conditions but had to continue hemodialysis at an out of hospital care. The anticoagulation with a vitamin K antagonist was initiated already in hospital and recommended to be maintained life-long.

## Discussion

Giant thrombi in the LA are quite rare, PubMed research showed only very few case reports, small studies and missing meaningful statistics [3–5]. In our local echocardiography database, this is the first case showing such a severe thrombotic condition of a THV. Thus, the true incidence is not known and there are no recommendations for treatment, especially in terms of proper anticoagulation. The options range from antiplatelet therapy (APT) to oral anticoagulation (OAC) or a combination of both. OAC can be realized by a Vitamin K antagonist or direct oral anticoagulants (DOACs). In the latest guidelines, for patients undergoing transcatheter aortic valve implantation (TAVI), a lifelong single APT is recommended, whereas an OAC is not recommended except there is another indication [1]. Of course, this recommendation cannot be taken as reference here since the pathophysiology and flow patterns are completely different in mitral compared to aortic valve position. When referring to the recommendation for bioprostheses in mitral position to prescribe a Vitamin K antagonist for the first 3 months [1], the following questions arise: first), are 3 months enough?; second), could we also use DOACs?; and third), what about patients with atrial fibrillation and successfully closed left atrial appendages?

In the process of decision making in the patient described here, we had to face all these questions and there are no sufficient data.

Since the course of the patients was uneventful after TMVR as long as apixaban was continued, DOACs seemed to be sufficient in this particular case. The question, whether initial treatment with a vitamin K antagonist instead of a DOAC would have made a difference, cannot be answered yet. Retrospectively, the turning point could have been the switch from DOAC to single APT in our case, may be having contributed to valve and LA thrombosis development within 2 months. This decision was made by focusing on sufficient surgical LAA closure and atrial fibrillation as indication for OAC against the background of an elevated risk for major bleeding with a HAS-BLED score of 3 points. Here, the presence of a relatively small and mildly stenotic THV in mitral position and the enlargement of the LA should be considered as arguments for a maintenance of OAC regardless of the condition, if LAA is closed or not. Irrespective of the presence of a THV in mitral position, success rates of surgical LAA closure strongly depend on the method used by the individual surgeon. Thus, performing a TOE should be performed in all cases and OAC should be stopped only in cases of proven success at most. In addition, one should have in mind that there are no definite data on the safety of anticoagulation discontinuation after surgical LAA closure until now [6, 7].

## Conclusion

Taken together, this case teaches us the difficulty of decision making in patients with atrial fibrillation undergoing TMVR and exhibiting both, high stroke and bleeding risk. OAC strategies should be evaluated very carefully and there should be valid arguments to discontinue OAC in an individual case. With the growing number of TMVR procedures performed nowadays, we will hopefully gain sufficient evidence for optimal management of these patients.

## Data Availability

Not applicable.

## Data availability

The datasets used and/or analysed during the current study available from the corresponding author on reasonable request.
